# The quark jet function for $k_T$-like variables in NNLO QCD

**DOI:** 10.1140/epjc/s10052-025-15048-2

**Published:** 2025-11-12

**Authors:** Luca Buonocore, Massimiliano Grazzini, Flavio Guadagni, Jürg Haag, Luca Rottoli

**Affiliations:** 1 Theoretical Physics Department, CERN, 1211 Geneva 23, Switzerland; 2 Physik Institut, Universität Zürich, 8057 Zürich, Switzerland; 3 Albert Einstein Center for Fundamental Physics, Institut für Theoretische Physik, Universität Bern, Sidlerstrasse 5, 3012 Bern, Switzerland; 4 Dipartimento di Fisica G. Occhialini, Università degli Studi di Milano-Bicocca and INFN, Sezione di Milano-Bicocca, Piazza della Scienza 3, 20126 Milan, Italy

## Abstract

The precise description of jet processes requires observables capable of efficiently capturing the dynamics of the energy flow in hadronic final states. We consider a class of transverse-momentum like resolution variables that smoothly describe the $n+1$ to *n* jet transition in multi-jet processes. We discuss a general method for the computation of the corresponding quark jet function at next-to-next-to-leading order in perturbative QCD. Rapidity divergences are regulated by using a time-like auxiliary vector. We present explicit results for a variant of $y_{23}$ in the *E*-scheme and in the WTA scheme.

## Introduction

Jet production at high-energy colliders is one of the most fundamental probes of Quantum Chromodynamics (QCD). At the Large Hadron Collider (LHC) jet studies are a staple in the precision physics programme. The vast amount of data collected so far provides important constraints in the extraction of parton distribution functions (PDFs) and in the determination of the strong coupling constant $\alpha _{\textrm{S}}$. This will be even more the case in the upcoming High-Luminosity phase of the LHC. Looking ahead, the accurate description of jet processes will be of high relevance at future $e^+e^-$ colliders.

At hadron colliders, $2 \rightarrow 2$ parton scattering constitutes the simplest jet production process. Next-to-next-to-leading order (NNLO) QCD corrections for single-jet inclusive and dijet observables have been obtained only recently [1–5]. The calculation of NNLO QCD corrections for $pp \rightarrow jjj$ production is among the most challenging and technically demanding computations undertaken to date [6]. Reaching NNLO accuracy to higher final-state multiplicities remains at the frontier of current computational capabilities. In $e^+e^-$ collisions, apart from dijet production, which is known up to next-to-next-to-next-to leading order ($\hbox {N}^3$LO) [7], NNLO predictions are available only for 3-jet production [8–10], highlighting the need for further advancements.

In regions of the phase space characterised by a large hierarchy of scales, jet observables become sensitive to the details of the QCD radiation. In such cases, all-order resummation becomes necessary to restore the predictive power of perturbation theory. While general-purpose parton showers provide a way to resum such contributions to all-order in perturbation theory, no general formalism exists for the resummation of observables beyond next-to-leading-logarithmic (NLL) accuracy, and only a relatively low number of observables are known at or beyond NNLL accuracy [11–18] in processes with three or more partons at the Born level.

A major obstacle in extending the description of jet observables to higher orders is the calculation of the NNLO ingredients entering the relevant resummation formulæ. In many cases, observables obey factorisation theorems that allow the organisation of the calculation by defining hard, soft, jet and beam functions. The hard function encodes the dynamics at the hard scale *Q* that characterises the underlying process. The soft function accounts for the emission of soft radiation from initial- and final-state partons. Beam functions describe the evolution of collinear partons along the beam directions, while jet functions capture the collinear dynamics of partons inside the jets. This organisation of the perturbative series in terms of hard, soft and collinear functions remains useful even in the absence of a factorisation theorem for the observable under consideration. A notable example is the two-jet rate $y_{23}$ in electron-positron collisions [19]. Since deviations from a fully factorized ansatz can be systematically computed order by order in perturbation theory, this strategy enables an efficient organisation of the fixed-order expansion of the observable at higher perturbative orders.

In this work, we focus on the computation of jet functions at next-to-next-to-leading order (NNLO). In particular, we consider the class of observables that scale as a transverse momentum in the limit where the radiation becomes soft and collinear to the jet direction corresponding to the leg $\ell$, i.e.

1 $$\begin{aligned} {\mathfrak {q}}\sim k_t^{(\ell )}. \end{aligned}$$

In the nomenclature of Soft-Collinear-Effective-Theory (SCET) [20–24], observables scaling as (1) belong to the class of $\hbox {SCET}_{\textrm{II}}$ variables.

In the case of observables like *N*-jettiness [25] or the transverse momentum of a colourless system (or heavy-quark pairs) and related variables, the perturbative knowledge of beam [26–35], soft [36–42] and jet [43–46] functions is available at NNLO and beyond. On the contrary, for the case of more general $k_T$-like variables in (1), which typically rely on the use of a jet clustering algorithm, the status of jet function calculations is less advanced due to the complications arising from the clustering history. A notable exception is inclusive jet production for the generalised $k_T$ jet algorithms. The two-loop anti-$k_T$ quark jet function for small jet radii has been computed in Ref. [47]. Aside from the TMD jet functions, which are partially known at NNLO [48, 49] and have also been employed for the variable defined in Ref. [50], state-of-the-art jet function calculations for generic $k_T$-like *n*-jet observables are currently limited to NLO [51–53]. It is worth emphasising that the knowledge of the beam, soft, and jet functions at a given perturbative order is not only essential for all-order resummation, but also enables fully differential fixed-order computations using slicing methods [54, 55], thereby broadening the scope of potential applications.

In this paper we present a semi-numerical strategy for the computation of the NNLO quark jet function for a $k_T$-like variable that smoothly captures the $n+1$ to *n* jet transition in $e^+e^-$ collisions. Our approach is fully general, and can be applied to any observable in the class (1). We illustrate the main steps of our method and we present explicit results for a variant of $y_{23}$ within the *E*-scheme and WTA [56] scheme. Related work has been independently presented in Refs. [57, 58].

It is well known that in the calculations of the perturbative jet, beam and soft function for $\hbox {SCET}_{\textrm{II}}$ observables one generally encounters divergent integrals that are not regulated by dimensional regularisation [59–63]. Such divergences require the introduction of an additional *rapidity* regulator, which is typically not necessary for $\hbox {SCET}_\textrm{I}$ observables. Several approaches to deal with this problem have been formulated [63–67]; in this paper we follow Ref. [33] and we regulate rapidity divergences through the introduction of a *timelike* reference vector.

In combination with a general study of the factorisation properties for this class of $k_T$-like observables [68], the results we are going to present constitute a building block for possible resummed computations for these observables and are directly relevant for NNLO calculations of multijet cross sections at high-energy colliders when these observables are used as slicing variables [69]. Our results may also have a relevance in the matching of fixed-order computations to parton shower simulations.

The paper is organised as follows. We introduce the framework for the computation of jet functions for $k_T$-like observables in Sect. 2. In Sect. 3 we give results for observables defined using the $k_T$ jet clustering algorithm for different jet recombination schemes. In Sect. 4 we summarise our findings and we draw our conclusions.

## The computation

In our computation we regularise both ultraviolet (UV) and infrared (IR) divergences by using conventional dimensional regularisation in $d=4-2\epsilon$ space-time dimensions. The $\textrm{SU}(N_c)$ QCD colour factors are $C_F=(N_c^2-1)/(2N_c)$, $C_A = N_c$, $T_R = 1/2$ and we consider $n_F$ massless quark flavours.

The bare cumulative jet function for a resolution variable ${\mathfrak {q}}$ is defined as

2 $$\begin{aligned}&{\mathcal {J}}^{ss^\prime }_{a}({\mathfrak {q}}_{\textrm{cut}})=\theta ({\mathfrak {q}}_{\textrm{cut}})\delta ^{ss^\prime }+\sum _{n=2}^\infty \sum _{{\mathfrak {A}}_{n}}2(2\pi )^{d-1}\!\nonumber \\&\quad \int \prod _{j=1}^n[dk_j]\delta \bigg (1-\sum _{j=1}^n z_j\bigg )\delta ^{(d-2)}\!\bigg ( \sum _{j=1}^n k_{j, \perp } \!\!\bigg )\nonumber \\&\quad \times \frac{\hat{{\mathcal {P}}} ^{ss^\prime }_{{\mathfrak {A}}_{n}}(k_1,k_2 \ldots k_n)}{S_{{\mathfrak {A}}_{n}}} \theta \!\Big ( {\mathfrak {q}}_{\textrm{cut}}- \tilde{{\mathfrak {q}}}\Big ) , \end{aligned}$$

where *a* is the parton flavour ($a=q,{{\bar{q}}},g$), ${\mathfrak {A}}_{n}$ is a multi-index containing the flavours of the *n* final-state QCD partons with momenta $k_1,k_2\ldots k_n$, $\tilde{{\mathfrak {q}}}=\tilde{{\mathfrak {q}}}(k_1,k_2\ldots k_n)$ is the collinear approximation of the resolution variable and $k_{j,\perp }$, $z_j$ are the (boost invariant) transverse momenta and momentum fractions involved in the multiparton collinear limit. The factor $1/S_{{\mathfrak {A}}_n}$ in Eq. (2) is a symmetry factor for identical particles and

3 $$\begin{aligned} {[}dk_j]=\frac{d^dk_j}{(2\pi )^{d-1}}\delta _+(k_j^2) \end{aligned}$$

is the phase space measure for massless particles.

The function $\hat{{\mathcal {P}}} _{{\mathfrak {A}}_n}(k_1,k_2\ldots k_n)$ in Eq. (2) controls the limit of the QCD matrix element squared $|{{{\mathcal {M}}}}(k_1,k_2\ldots k_n\ldots )|^2$ in which *n* final state partons become collinear

4 $$\begin{aligned} \left| {{{\mathcal {M}}}}_{a_1,a_2..a_n\ldots }(k_1,k_2\ldots k_n\ldots )\right| ^2\simeq {{{\mathcal {T}}}}_a^{ss^\prime }(p\ldots ) \hat{{\mathcal {P}}} _{a_1,a_2\ldots a_n}^{ss^\prime } \, , \end{aligned}$$

${{{\mathcal {T}}}}_a^{ss^\prime }(p\ldots )$ being the spin polarisation tensor obtained by replacing the collinear partons with the parent parton *a*, which carries the flavour and momentum of the system $a_1+a_2+\cdots a_n$ in the collinear limit. The function $\hat{{\mathcal {P}}} _{a_1,a_2\ldots a_n}^{ss^\prime }$ is related to the customary collinear splitting kernels through the relation

5 $$\begin{aligned} \hat{{\mathcal {P}}} _{a_1,a_2\ldots a_n}^{ss^\prime } =\left( \frac{8\pi \alpha _{\textrm{S}}^u\mu _0^{2\epsilon }}{s_{1\dots n}}\right) ^{n-1}{{\hat{P}}}^{ss'}_{a_1,a_2\ldots a_n}, \end{aligned}$$

where $s_{1\dots n}=(k_1+k_2+\dots k_n)^2$. The functions ${{\hat{P}}}_{a_1,a_2\ldots a_n}$ have the perturbative expansion in the bare coupling $\alpha _0$

6 $$\begin{aligned} {{\hat{P}}}^{ss'}_{a_1,a_2\ldots a_n}=\sum _{l=0}^\infty \left( \frac{\alpha _0}{\pi }\right) ^l\left( \frac{\mu ^2}{s_{1\ldots n}}\right) ^{l \epsilon }{{\hat{P}}}_{a_1,a_2\ldots a_n}^{(l)ss'} , \end{aligned}$$

where ${{\hat{P}}}^{(0)}$ is the tree-level contribution, ${{\hat{P}}}^{(1)}$ the one-loop correction, and so forth. The jet function ${{\mathcal {J}}}_a^{ss^\prime }({\mathfrak {q}}_{\textrm{cut}})$ is in general a matrix in the spin indices *s* and $s^\prime$ of the parton *a*. In the quark case, spin correlations are absent, and the matrix is diagonal

7 $$\begin{aligned} {{\mathcal {J}}}_q^{ss^\prime }({\mathfrak {q}}_{\textrm{cut}})={{\mathcal {J}}}_q({\mathfrak {q}}_{\textrm{cut}})\delta ^{ss^\prime }. \end{aligned}$$

The perturbative expansion of ${{\mathcal {J}}}_{q}({\mathfrak {q}}_{\textrm{cut}})$ reads

8 $$\begin{aligned} {{\mathcal {J}}}_{q}({\mathfrak {q}}_{\textrm{cut}})=\theta ({\mathfrak {q}}_{\textrm{cut}}) +\sum _{n=1}^\infty \left( \frac{\alpha _0}{\pi } \right) ^n\left( \frac{\mu ^2}{{\mathfrak {q}}_{\textrm{cut}}^2}\right) ^{n\epsilon }{{\mathcal {J}}}_{q}^{(n)}({\mathfrak {q}}_{\textrm{cut}}). \end{aligned}$$

The bare coupling $\alpha _0=\alpha _0(\mu )$ is related to the (dimensionless) unrenormalised coupling $g_{\mathrm S}^u$ in the QCD Lagrangian by the relation

9 $$\begin{aligned} \alpha _{\textrm{S}}^u\mu _0^{2\epsilon } S_\epsilon =\alpha _0(\mu ) \mu ^{2\epsilon } \end{aligned}$$

with $\alpha _{\textrm{S}}^u=(g_{\mathrm S}^u)^2/(4\pi )$, and $S_\epsilon$ is the customary spherical factor $S_\epsilon =(4\pi )^\epsilon e^{-\epsilon \gamma _E}$.

The computation of the jet function in Eq. (2) is generally affected by rapidity divergences [59–63], which manifest themselves as singularities as $z_i\rightarrow 0$ that are not regulated by dimensional regularisation. In this paper we treat rapidity divergences by using a *time-like* auxiliary vector *N* to define the momentum fractions. This regularisation scheme, that we dub “$z_N$ prescription”, has been introduced in Ref. [33]^1^. The momentum fraction $z_N$ for a parton with momentum *k* reads

10 $$\begin{aligned} z_N=\frac{k\cdot N}{p\cdot N}=z+\frac{N^2 k_\perp ^2}{(2p\cdot N)^2 z} , \end{aligned}$$

where *p* is the collinear direction and $k_\perp$ is the transverse momentum involved in the collinear splitting. In the strict collinear limit the term proportional to $k_\perp ^2$ in Eq. (10) is subleading. The presence of $N^2>0$ in Eq. (10) prevents factors $1/z_N$ in the splitting kernels from becoming singular and ensures that the integrals in Eq. (2) are well-defined. Due to the $z_{N}$ prescription, the integral associated with the soft endpoint of the splitting kernel is no longer scaleless. As a result, the procedure generates a non-vanishing *zero-bin* contribution, which we combine with the soft function to avoid double counting. A general definition of a *subtracted* soft function in which the zero-bin contributions from the jet- and beam-functions are removed is provided in Ref. [68]. We note that in our calculation the replacement $z\rightarrow z_N$ is implemented only in the singular term of the relevant splitting kernels, as described below. We denote the perturbative coefficients of the bare jet function with this regularisation by ${\mathcal {J}}^{(n)}_{N,q}$.

We start from the NLO computation. The only quark-initiated splitting at this order is $q\rightarrow gq$. The relevant splitting kernel is

11 $$\begin{aligned} {{\hat{P}}}^{(0)}_{gq}(z)=C_F\left[ \frac{1+(1-z)^2}{z}-\epsilon z\right] . \end{aligned}$$

The regularised kernel ${{\hat{P}}}^{(0)}_{N,gq}(z)$ is obtained from ${{\hat{P}}}^{(0)}_{gq}(z)$ by isolating the 1/*z* singular term and replacing *z* with $z_N$ therein:

12 $$\begin{aligned} {{\hat{P}}}^{(0)}_{N,gq}(z)=C_F\left[ \frac{2}{z_N}-2+(1-\epsilon )z\right] . \end{aligned}$$

From Eqs. (2) and (8) we have

13 $$\begin{aligned} {\mathcal {J}}^{(1)}_{N,q}({\mathfrak {q}}_{\textrm{cut}}) = 4\, {\mathfrak {q}}_{\textrm{cut}}^{2\epsilon }\frac{e^{\epsilon \gamma _E}}{\Gamma (1-\epsilon )}\! \int \! d\Phi _2^{(c)} \frac{{{\hat{P}}}^{(0)}_{N,gq}(z_1)}{2 k_1\cdot k_2} \theta ({\mathfrak {q}}_{\textrm{cut}}- \tilde{{\mathfrak {q}}}), \end{aligned}$$

where we defined the collinear phase space

14 $$\begin{aligned} d\Phi _n^{(c)}= &   \frac{1}{\left( \Omega _{2-2\epsilon } \right) ^{n-1}}\left( \prod _{j=1}^n d^dk_j \delta _+(k_j^2)\right) \nonumber \\  &   \quad \times \delta \left( 1-\sum _{j=1}^n z_j\right) \delta ^{(d-2)}\left( \sum _{j=1}^n\vec {k}_{j,\perp }\right) \,. \end{aligned}$$

In the following, we focus on the particular case where the variable fulfils the condition $\tilde{{\mathfrak {q}}}=k_\perp$ 2 in the two-parton collinear limit. Then, we can further manipulate the previous equation as

15 $$\begin{aligned}  &   {\mathcal {J}}^{(1)}_{N,q}({\mathfrak {q}}_{\textrm{cut}})\nonumber \\  &   \quad ={\mathfrak {q}}_{\textrm{cut}}^{2\epsilon }\frac{e^{\epsilon \gamma _E}}{\Gamma (1-\epsilon )}\int _0^1 dz \int _0^{{\mathfrak {q}}_{\textrm{cut}}} dk_\perp k_\perp ^{-1-2\epsilon } {{\hat{P}}}^{(0)}_{N,gq}(z). \end{aligned}$$

The final integration can be carried out by using the identity

16 $$\begin{aligned} \frac{1}{z_N}=\left( \frac{1}{z}\right) _+-\frac{1}{2}\delta (z)\ln \frac{N^2 k^2_\perp }{(2p\cdot N)^2}+{{{\mathcal {O}}}}\left( \frac{k_\perp \sqrt{N^2}}{2p\cdot N}\right) \end{aligned}$$

and we obtain

17 $$\begin{aligned} {{\mathcal {J}}}_{N, q}^{(1)}=\frac{e^{\epsilon \gamma _E}}{\Gamma (1-\epsilon )} C_F\left[ \frac{1}{2\epsilon ^2}+\frac{L_N}{\epsilon }+\frac{3}{4\epsilon }+\frac{1}{4}\right] \, , \end{aligned}$$

where we have defined

18 $$\begin{aligned} L_N=\ln \left( \frac{\sqrt{N^2}{\mathfrak {q}}_{\textrm{cut}}}{2p\cdot N}\right) . \end{aligned}$$

At NNLO the quark jet function receives contributions from the $q\rightarrow gq$ splitting at one-loop order and from all the possible tree-level triple collinear splittings $q\rightarrow {{\bar{q}}}^\prime q^\prime q$, $q\rightarrow {{\bar{q}}}qq$, $q\rightarrow ggq$. The one-loop contribution is controlled by the $q\rightarrow gq$ splitting kernel, which reads [70, 71]

19 $$\begin{aligned}&{{\hat{P}}}_{gq}^{(1)}(z)=\frac{1}{2}c_P\cos (\pi \epsilon )\Bigg \{C_F(C_A-C_F)\frac{1-\epsilon z}{1-2\epsilon }\nonumber \\&\quad +{{\hat{P}}}^{(0)}_{gq}(z)\left( -\frac{1}{\epsilon ^2}\right) \Bigg [C_A\,{}_2F_1\left( 1,-\epsilon ,1-\epsilon ,1-\frac{1}{z}\right) \nonumber \\&\quad -2(C_F-\frac{1}{2}C_A)\left( 1-{}_2F_1\left( 1,-\epsilon ,1-\epsilon ,-\frac{z}{1-z}\right) \right) \Bigg ]\Bigg \}, \end{aligned}$$

where

20 $$\begin{aligned} c_P&=e^{\epsilon \gamma _E}\frac{\Gamma ^2(1-\epsilon )\Gamma (1+\epsilon )}{\Gamma (1-2\epsilon )}\nonumber \\&=1-\frac{\pi ^2}{12}\epsilon ^2-\frac{7}{3}\zeta _3 \epsilon ^3-\frac{47}{1440}\pi ^4 \epsilon ^4+{{{\mathcal {O}}}}(\epsilon ^5). \end{aligned}$$

The regularised version of the one-loop splitting kernel ${{\hat{P}}}^{(1)}_{N,gq}(z)$ is obtained by replacing the tree level kernel ${{\hat{P}}}^{(0)}_{gq}(z)$ in the first line of Eq. (19) with ${{\hat{P}}}^{(0)}_{N,gq}(z)$^3^. The ensuing contribution to the jet function can be straightforwardly evaluated by replacing the tree-level kernel ${{\hat{P}}}^{(0)}_{N,gq}(z)$ with its one-loop correction ${{\hat{P}}}^{(1)}_{N,gq}(z)$ in Eq. (13).

The computation of the tree-level contributions to ${\mathcal {J}}^{(2)}_{N,q}$ is driven by the $1\rightarrow 3$ collinear splittings [72–74]. The collinear approximation is now defined in terms of two momentum fractions $z_{i}$ and $z_{j}$. Consequently, the structure of rapidity divergences becomes more intricate, and the extension of the $z_{N}$ prescription correspondingly more subtle. Our strategy relies on analysing the divergences that can occur in the splitting kernels, i.e., at the level of the squared matrix elements. For configurations characterised by strongly-ordered independent emissions, rapidity divergences arise in the limit where one or both momentum fractions vanish, $z_{i}\rightarrow 0$ and/or $z_{j}\rightarrow 0$. In contrast, for configurations involving correlated emissions, rapidity divergences occur only when the sum of the momentum fractions vanishes, $z_{i}+z_{j}\rightarrow 0$. The former case displays a more intricate structure due to the overlap of the two limits. Since we only require that the $z_{N}$ replacement yields finite integrals, a certain freedom remains in the precise definition of the regularised kernels, which is compensated by different zero-bin contributions. In the following, we present our analysis for all the $1\rightarrow 3$ splittings relevant to the quark jet function, and provide the explicit expressions of the corresponding $z_{N}$-regularised kernels.

We start from the different flavour $q\rightarrow {{\bar{q}}}'q'q$ term. The corresponding splitting kernel reads

21 $$\begin{aligned} {{\hat{P}}}^{(0)}_{\bar{q}'_1 q'_2 q_3}&= \frac{1}{2}C_FT_R \frac{s_{123}}{s_{12}} \bigg [\bigg ( -\frac{t_{12,3}^2}{s_{12}s_{123}} + \frac{4 z_3 + (z_1-z_2)^2}{z_1+z_2}\bigg )\nonumber \\&\quad + (1-2\epsilon ) \bigg ( z_1+z_2 - \frac{s_{12}}{s_{123}}\bigg ) \bigg ], \end{aligned}$$

where

22 $$\begin{aligned} t_{ij,k}=2\frac{z_i s_{jk}-z_j s_{ik}}{z_i+z_j}+\frac{z_i-z_j}{z_i+z_j}s_{ij}. \end{aligned}$$

In this case the rapidity divergence appears only when the sum of the quark and antiquark momentum fractions $z_1+z_2$ vanishes. The modified splitting kernel is obtained from Eq. (21) as

23 $$\begin{aligned} {{\hat{P}}}^{(0)}_{N,\bar{q}'_1 q'_2 q_3}= &   \frac{1}{2}C_FT_R \frac{s_{123}}{s_{12}} \bigg [ \frac{1}{z_{1,N} + z_{2,N}}\bigg ( -\frac{(z_1+z_2)t_{12,3}^2}{s_{12}s_{123}} \nonumber \\  &   + 4 z_3 + (z_1-z_2)^2\bigg ) \nonumber \\  &   + (1-2\epsilon ) \bigg ( z_1+z_2 - \frac{s_{12}}{s_{123}}\bigg ) \bigg ]. \end{aligned}$$

The related contribution to the jet function is

24 $$\begin{aligned} {{\mathcal {J}}}_{N, q}^{(2)}({\mathfrak {q}}_{\textrm{cut}})\Big |_{C_FT_R}&=8 {\mathfrak {q}}_{\textrm{cut}}^{4\epsilon }\frac{e^{2\epsilon \gamma _E}}{\Gamma ^2(1-\epsilon )}\nonumber \\&\quad \times \int d\Phi _3^{(c)}\frac{{{\hat{P}}}^{(0)}_{N,\bar{q}'_1q'_2q_3}}{s_{123}^2} \theta ({\mathfrak {q}}_{\textrm{cut}}-\tilde{{\mathfrak {q}}}). \end{aligned}$$

We find it useful to employ the variables defined in Ref. [75]:

25 $$\begin{aligned}  &   a = \frac{z_1 k_{2,\perp }}{z_2k_{1,\perp }}, \quad b=\frac{k_{1,\perp }}{k_{2,\perp }}, \quad z=z_1+z_2,\nonumber \\  &   m_T = \sqrt{z\left( \frac{k_{1,\perp }^2}{z_1} + \frac{k_{2,\perp }^2}{z_2} \right) }, \quad x_{12} = \frac{1-\cos \varphi _{12}}{2} \,. \end{aligned}$$

The variable *a* represents the rapidity difference between $k_1$ and $k_2$, *z* is the momentum fraction of the momentum $k_1+k_2$, while $m_T$ is its transverse mass, and $\varphi _{12}$ is the angle between the $(d-2)$-dimensional vectors $\vec {k}_{1,\perp }$ and $\vec {k}_{2,\perp }$. The parametrization has only one dimensionful variable, $m_T$, and $\tilde{{\mathfrak {q}}}$ can be written as

26 $$\begin{aligned} \tilde{{\mathfrak {q}}}^2 = m^2_T F(a,b,z,x_{12}), \end{aligned}$$

where the dimensionless function $F(a,b,z,x_{12})$ specifies the observable. In the new variables, we can write

27 $$\begin{aligned} z_{1,N} + z_{2,N}=z+\frac{m_T^2 N^2}{z(2p\cdot N)^2}, \end{aligned}$$

and we can use Eq. (16) to extract the leading-power contribution from the regularised splitting kernel. The integration over the dimensionful variable $m_T$ is straightforward, and the rest of the calculation can be organised by remapping the remaining variables onto the unit hypercube, with suitable changes of variables to avoid overlapping singularities. At this point, the integrand can be expanded in $\epsilon$ and the final integrations are carried out numerically.

The contribution of the $q\rightarrow {{\bar{q}}}qq$ splitting is obtained from the $q\rightarrow {{\bar{q}}}'q'q$ by adding the interference term

28 $$\begin{aligned} {{\hat{P}}}^{(0)}_{\bar{q}_1 q_2 q_3} = [{{\hat{P}}}^{(0)}_{\bar{q}'_1 q'_2 q_3 } + (2\leftrightarrow 3)] + {{\hat{P}}}_{\bar{q}_1 q_2 q_3 }^{(0)(\text {id})} , \end{aligned}$$

where

29 $$\begin{aligned} {{\hat{P}}}_{\bar{q}_1q_2q_3}^{(0)(\text {id})}&= C_F\left( C_F -\frac{1}{2}C_A \right) \left\{ (1-\epsilon ) \left( \frac{2 s_{23}}{s_{12}}-\epsilon \right) \right. \end{aligned}$$

30 $$\begin{aligned}&\quad +\frac{s_{123}}{s_{12}}\left[ \frac{1+z_1^2}{1-z_2}-\frac{2z_2}{1-z_3} \right. \nonumber \\&\left. \quad - \epsilon \left( \frac{(1\!-\!z_3)^2}{1\!-\!z_2} \!+\!1\!+\!z_1\!-\!\frac{2z_2}{1\!-\!z_3}\right) \!-\!\epsilon ^2 (1-z_3) \right] \end{aligned}$$

31 $$\begin{aligned}&\quad -\frac{s_{123}^2}{s_{12}s_{13}}\frac{z_1}{2}\left[ \frac{1+z_1^2}{(1-z_2)(1-z_3)}\right. \nonumber \\&\quad \left. \left. - \epsilon \left( 1+2\frac{1-z_2}{1-z_3} \right) - \epsilon ^2 \right] \right\} + (2\leftrightarrow 3). \end{aligned}$$

The splitting kernel ${{\hat{P}}}_{\bar{q}_1q_2q_3}^{(0)(\text {id})}$ does not exhibit rapidity divergences and therefore we do not define a $z_N$-regularised version of it. The computation can be carried out with the parametrisation of Eq. (25) with no additional complications.

We now move to the $q\rightarrow ggq$ contribution, which can be organised into an abelian and a non-abelian part:

32 $$\begin{aligned} {{\hat{P}}}^{(0)}_{g_1 g_2 q_3} = C_F^2 {{\hat{P}}}_{g_1 g_2 q_3}^{(0)(\text {ab})} + C_F C_A {{\hat{P}}}_{g_1 g_2 q_3}^{(0)(\text {nab})}. \end{aligned}$$

The non-abelian $q\rightarrow ggq$ kernel reads

33 $$\begin{aligned} \begin{aligned}&P^{(0)(\mathrm nab)}_{g_1g_2q_3} = \left\{ (1-\epsilon )\left( \frac{t_{12,3}^2}{4s_{12}^2} + \frac{1}{4} - \frac{\epsilon }{2}\right) \right. \\&\quad + \frac{s_{123}^2}{2s_{12}s_{13}} \left[ \frac{(1-z_3)^2(1-\epsilon ) + 2z_3}{z_2} \right. \\&\quad \left. +\frac{z_2^2(1-\epsilon )+2(1-z_2)}{1-z_3} \right] \\&\quad - \frac{s_{123}^2}{4s_{13}s_{23}}z_3 \left[ \frac{(1-z_3)^2(1-\epsilon )+2z_3}{z_1z_2} + \epsilon (1-\epsilon ) \right] \\&\quad + \frac{s_{123}}{2s_{12}} \left[ (1-\epsilon ) \frac{z_1(2-2z_1+z_1^2)-z_2(6-6z_2+z_2^2)}{z_2(1-z_3)} \right. \\&\left. \quad + 2\epsilon \frac{z_3(z_1-2z_2)-z_2}{z_2(1-z_3)} \right] \\&\quad + \frac{s_{123}}{2s_{13}} \left[ (1-\epsilon ) \frac{(1-z_2)^3+z_3^2-z_2}{z_2(1-z_3)}\right. \\&\quad - \epsilon \left( \frac{2(1-z_2)(z_2-z_3)}{z_2(1-z_3)}-z_1+z_2 \right) \\&\quad -\frac{z_3(1-z_1)+(1-z_2)^3}{z_1z_2} \\&\quad \left. \left. + \epsilon (1-z_2)\left( \frac{z_1^2+z_2^2}{z_1z_2}-\epsilon \right) \right] \right\} + (1\leftrightarrow 2). \end{aligned} \end{aligned}$$

In this case, like the $q\rightarrow \bar{q}'q'q$ case, the emissions are correlated, and it is possible to check that the rapidity divergence appears only when the sum $z_1+z_2$ vanishes. Therefore, we define the $z_N$-regularised version of this splitting kernel by only modifying the terms that are singular when the sum of the two momentum fraction vanishes:

34 $$\begin{aligned} \begin{aligned} {{\hat{P}}}_{N,g_1g_2q_3}^{(0)\mathrm {(nab)}}&={{\hat{P}}}_{g_1g_2q_3}^{(0)\mathrm {(nab)}} - \bigg (1-\frac{z_1+z_2}{z_{N,1}+z_{N,2}} \bigg ) \\&\quad \times \bigg [ \frac{(1-\epsilon )(z_1s_{23}-z_2s_{13})^2}{(z_1+z_2)^2 s_{12}^2}\\&\quad +\frac{(z_1+2z_2)s_{123}^2}{z_2(z_1+z_2)s_{12}s_{13}}-\frac{s_{123}^2}{2z_1 z_2 s_{13} s_{23}} \\&\quad +\! \frac{(z_1-3z_2)s_{123}}{z_2(z_1\!+\!z_2)s_{12}} \!-\! \frac{s_{123}}{z_1(z_1\!+\!z_2)s_{13}} \!+\! (1 \!\leftrightarrow \!2)\bigg ]. \end{aligned} \end{aligned}$$

The corresponding calculation can be carried out similarly to what was done for $C_F T_R$. The only difference is that in this case there is an additional pole due to the configuration in which one of the two emitted gluons is soft. This configuration, however, does not lead to additional complications.

The most intricate part of the computation is the evaluation of the abelian $q\rightarrow ggq$ contribution. The corresponding splitting kernel is:

35 $$\begin{aligned} \begin{aligned} {{\hat{P}}}_{g_1 g_2 q_3}^{(0)(\text {ab})}&= \left\{ \frac{s_{123}^2}{2s_{13}s_{23}}z_3 \left[ \frac{1+z_3^2}{z_1z_2} -\epsilon \frac{z_1^2+z_2^2}{z_1z_2}- \epsilon (1+\epsilon )\right] \right. \\&\quad +\frac{s_{123}}{s_{13}}\left[ \frac{z_3(1-z_1)+(1-z_2)^3}{z_1z_2}+\epsilon ^2 (1+z_3)\right. \\&\quad \left. -\epsilon (z_1^2+z_1z_2+z_2^2)\frac{1-z_2}{z_1z_2} \right] \\&\quad \left. +(1-\epsilon ) \left[ \epsilon -(1-\epsilon ) \frac{s_{23}}{s_{13}}\right] \right\} + (1\leftrightarrow 2). \end{aligned} \end{aligned}$$

In this case the emitted gluons are uncorrelated, and the rapidity divergences appear also when only one of the two momentum fractions $z_1$ and $z_2$ vanishes. To define the $z_N$-regularised version of the abelian $q\rightarrow ggq$ splitting, we isolate its strongly-ordered limit by defining:

36 $$\begin{aligned} {{\hat{P}}}_{g_1 g_2 q_3}^{(0)(\textrm{ab})} = {{\hat{P}}}_{g_1 g_2 q_3}^{(0)\mathrm {(ab),S.O.}}+{{\hat{P}}}_{g_2 g_1 q_3}^{(0)\mathrm {(ab),S.O.}} + R_{g_1 g_2 q_3}^{(0)(\textrm{ab})} , \end{aligned}$$

where the strongly-ordered term reads

37 $$\begin{aligned} {{\hat{P}}}_{g_1 g_2 q_3}^{(0)\mathrm {(ab),S.O.}} =\frac{s_{123}}{s_{23}} {{\hat{P}}}^{(0)}_{gq}(z_1){{\hat{P}}}^{(0)}_{gq}\bigg (\frac{z_2}{z_2+z_3}\bigg ). \end{aligned}$$

The remainder $R_{g_1 g_2 q_3}^{(0)(\textrm{ab})}$ does not feature rapidity divergences and therefore does not need regularisation. The strongly-ordered contribution ${{\hat{P}}}_{g_1 g_2 q_3}^{(0)\mathrm {(ab),S.O.}}$ is regularised by substituting the NLO splitting kernels with their $z_N$ versions:

38 $$\begin{aligned} {{\hat{P}}}_{N,g_1 g_2 q_3}^{(0)\mathrm {(ab),S.O.}} =\frac{s_{123}}{s_{23}} {{\hat{P}}}^{(0)}_{N,gq}(z_1){{\hat{P}}}^{(0)}_{N,gq}\bigg (\frac{z_2}{z_2+z_3}\bigg ). \end{aligned}$$

The contribution coming from the remainder $R_{g_1g_2q_3}^{(0)(\textrm{ab})}$ can be computed with the strategy employed for the $C_F T_R$ and $C_F C_A$ terms. The most complicated part of our calculation is the integration of the strongly-ordered term. For this contribution we find it useful to employ the following phase space parametrization. We introduce the variables

39 $$\begin{aligned} \tilde{z}_2 = \frac{z_2}{z_2+z_3}, \quad \vec {k}_{23,\perp } = \frac{z_3 \vec {k}_{2,\perp } - z_2 \vec {k}_{3,\perp }}{z_2+z_3}. \end{aligned}$$

The variable $\tilde{z}_2$ is the momentum fraction associated with the splitting $q\rightarrow g_2 q_3$ and $\vec {k}_{23,\perp }$ is the relative transverse momentum of the partons 2 and 3, divided by their total momentum fraction. With these variables, we can write

40 $$\begin{aligned}  &   z_{N,1}=z_1+\frac{N^2 k_{1,\perp }^2}{(2p\cdot N)^2 z_1}\nonumber \\  &   \quad \tilde{z}_{N,2}=\tilde{z}_{2}+\frac{N^2 k_{23,\perp }^2}{(2p\cdot N)^2 \left( 1-z_1 \right) ^2\tilde{z}_2}+\dots , \end{aligned}$$

where the dots represent terms that are subleading in all relevant limits. We write the phase space in terms of the variables

41 $$\begin{aligned}&z_1, \quad \tilde{z}_2,\quad y \equiv \min \bigg \{\frac{k_{1,\perp }}{k_{23,\perp }}, \frac{k_{23,\perp }}{k_{1,\perp }}\bigg \}, \nonumber \\&\quad k_\perp \equiv \max \{k_{1,\perp },k_{23,\perp }\}, \quad \cos \varphi \equiv \frac{\vec {k}_{1,\perp }\cdot \vec {k}_{23,\perp }}{k_{1,\perp } k_{23,\perp }}. \end{aligned}$$

The main difficulty in integrating the strongly-ordered splitting function arises from the presence of rapidity divergences in the individual limits $z_1 \rightarrow 0$ and $z_2 \rightarrow 0$, not only in the combined limit $z_1 + z_2 \rightarrow 0$. To overcome this complication, we divide the integration of $P_{N,g_1g_2q_3}^{(0),\mathrm {(ab)S.O.}}$ into two pieces:

42 $$\begin{aligned}&\int d\Phi _3^{(c)}\frac{P_{N,\,g_1g_2q_3}^{(0),\mathrm (ab)S.O.}}{s_{123}^2}\theta ({\mathfrak {q}}_{\textrm{cut}}- \tilde{{\mathfrak {q}}}) \nonumber \\&\quad = \int d\Phi _3^{(c)}\frac{P_{N,\,g_1g_2q_3}^{(0),\mathrm (ab)S.O.}}{s_{123}^2}\theta ({\mathfrak {q}}_{\textrm{cut}}- k_\perp ) \nonumber \\&\quad + \int d\Phi _3^{(c)}\frac{P_{N,\,g_1g_2q_3}^{(0),\mathrm (ab)S.O.}}{s_{123}^2}\theta _{\textrm{sub}}, \end{aligned}$$

where we defined the subtracted Heaviside function

43 $$\begin{aligned} \theta _{\textrm{sub}} = \theta ({\mathfrak {q}}_{\textrm{cut}}- \tilde{{\mathfrak {q}}}) - \theta ({\mathfrak {q}}_{\textrm{cut}}- k_\perp ). \end{aligned}$$

We refer to the first term on the right-hand side of Eq. (42) as the *endpoint term*, and to the second as the *subtracted term*. The endpoint term is common to all variables belonging to the class considered in this paper. The subtracted term is free of poles, since $\theta _{\textrm{sub}}$ acts as a counterterm that regulates all the singularities of $P_{N,\,g_1g_2q_3}^{(0),\mathrm {(ab)S.O.}}$, but still contains terms proportional to powers of $L_N$.

To integrate the subtracted term, we separate contributions that contain at most one regularised momentum fraction, and contributions that contain the product $1/(z_{1,N}\tilde{z}_{2,N})$. In the integrals in which only one of the momentum fractions is replaced with its $z_N$ version, the calculation can be carried out by using identities similar to Eq. (16). In the integrals in which both momentum fractions need to be regularised by the $z_N$ prescription, we cannot simply use Eq. (16) on both momentum fractions separately. Instead, we need to perform a uniform expansion of the product $1/(z_{1,N}\tilde{z}_{2,N})$ in the limit in which both gluons become soft. We provide the relevant expansion in Appendix A in Eq. (55).

The endpoint term is the only one leading to $\epsilon$ poles. Furthermore, it contains additional divergences in the strongly-ordered limit, $y \rightarrow 0$, that overlap with the rapidity divergences. This prevents us from directly applying the expansions in Eqs. (16) and (55). However, since the $\theta$-function in the endpoint term cuts on $k_\perp$ rather than $\tilde{{\mathfrak {q}}}$, we can calculate the endpoint term once and for all using for example sector decomposition techniques [76]. The explicit result for the endpoint contribution is presented in Sect. 3.

## Results

We apply the general strategy outlined in the previous section to the computation of the jet function for a generic *n*-jet resolution variable defined by using a recursive recombination jet algorithm with the distance

44 $$\begin{aligned} d_{ij}^2 = \frac{E_i^2 E_j^2}{(E_i+E_j)^2} 2(1-\cos \theta _{ij})\, . \end{aligned}$$

This is a possible variant of the distance used in the $k_T$ algorithm for $e^+e^-$ collisions [77], which makes the calculations slightly simpler. In particular, for a single emission the variable approaches the transverse momentum relative to the jet direction in the two-parton collinear limit, and, thus, at NLO the jet function is given by Eq. (17).^4^ We run the recursive recombination on a generic $n+k$ parton system until $n+1$ pseudopartons are left. We then define ${\mathfrak {q}}=\min \{d_{ij}\}$. We consider two recombination schemes:

- *E*-scheme, where the momentum $k_{ij}$ obtained recombining particles with momenta $k_i$ and $k_j$ is
  45 $$\begin{aligned} k_{ij} = k_i + k_j. \end{aligned}$$
- Winner-take-all (WTA) scheme [56], where the momentum of the recombined particle is
  46 $$\begin{aligned} k_{ij} = (E_i+E_j)\left( \frac{k_i}{E_i}\theta (E_i-E_j) + \frac{k_j}{E_j} \theta (E_j-E_i)\right) . \end{aligned}$$

In the *E*-scheme, the recombined momentum is simply the sum of the original momenta, and in general it is massive. In the WTA scheme, the recombined momentum is massless and it has the direction of the more energetic pseudo-parton. The variable ${\mathfrak {q}}$ belongs to the general class of transverse-momentum variables considered in Ref. [52], and dubbed $k_T^{\text {ness}}$.

In the parametrization of Eq. (25), the dimensionless observable function $F(a,b,z,x_{12})$ reads

47 $$\begin{aligned}&F(a,b,z,x_{12}) = \Theta _{12} F_{12}(a,b,z,x_{12})\nonumber \\&\quad +\Theta _{13} F_{13}(a,b,z,x_{12})+\Theta _{23} F_{23}(a,b,z,x_{12}), \end{aligned}$$

where $\Theta _{ij}\equiv \theta (d_{ik}-d_{ij})\theta (d_{jk}-d_{ij})$ specifies the phase space region in which partons *i* and *j* are clustered first. In the *E*-scheme, the functions $F_{ij}(a,b,z,x_{12})$ read

48 $$\begin{aligned}&F_{12}(a,b,z,x_{12}) = \frac{a[(1+b)^2-4bx_{12}]}{(a+b)(1+ab)},\nonumber \\&F_{13}(a,b,z,x_{12}) = \frac{a}{(a+b)(1+ab)}, \nonumber \\&F_{23}(a,b,z,x_{12}) = \frac{ab^2}{(a+b)(1+ab)}. \end{aligned}$$

In the WTA scheme, they are given by

49 $$\begin{aligned}&F_{12}^{\textrm{WTA}}(a,b,z,x_{12}) = \frac{a}{(a+b)(1+ab)}\nonumber \\&\quad \times \Big [ (1+ab)^2 - 2b(1+ab)z(-1+a+2x_{12})\nonumber \\&\quad + b^2 \big ((1-a)^2 + 4 a x_{12}\big ) z^2 \Big ], \nonumber \\&F_{13}^{\textrm{WTA}}(a,b,z,x_{12}) = \frac{(1+ab-z)^2}{a(a+b)(1+ab)^5(1-z)^2} \nonumber \\&\quad \times \Bigg \{ \bigg [ a^2 (1+a^2 b^2(1-z)^2 + 2 b z -4 b x_{12} z \nonumber \\&\quad + b^2 z^2 + 2 a b (z-1) (-1+b(-1+2x_{12})z) \bigg ] \nonumber \\&\quad \times \theta \big ( 1+ab-z-2abz \big ) \nonumber \\&\quad + \bigg [(1+ab)^2 \big (1+a^2 + a(-2+4x_{12})\big ) (1-z)^2 \bigg ] \nonumber \\&\qquad \theta \big (-1-ab+z+2abz\big ) \Bigg \}, \nonumber \\&F_{23}^{\textrm{WTA}}(a,b,z,x_{12}) = \frac{ab^2(-1+ab(-1+z))^2}{(a+b)(1+ab)^5(1-z)^2} \nonumber \\&\quad \times \Bigg \{ \bigg [(1+ab)^2 (1+a^2 +a(-2+4x_{12}))(1-z)^2 \bigg ] \nonumber \\&\quad \times \theta \big (-1-ab+2z+abz\big ) \nonumber \\&\quad + \bigg [ (-1+z)^2 -2a(-1+z)(b+z-2x_{12}z) + \nonumber \\&\quad + a^2 (b^2 + b(2-4x_{12})z + z^2 ) \bigg ] \nonumber \\&\quad \times \theta \big (1+ab-2z-abz \big ) \Bigg \}. \end{aligned}$$

We are now ready to present our results. The NNLO quark jet function can be written as

50 $$\begin{aligned} {{\mathcal {J}}}_{N,q}^{(2)} = L_N^2 \sum _{k=0}^2 \frac{D_k}{\epsilon ^k} + L_N \sum _{k=0}^3 \frac{A_k}{\epsilon ^k} + \sum _{k=0}^4 \frac{B_k}{\epsilon ^k}, \end{aligned}$$

where $L_N$ is defined in Eq. (18). The coefficients $D_k$, $A_k$ and $B_k$ for $k\ge 1$ do not depend on the recombination scheme and read

51 $$\begin{aligned} D_2&= \frac{C_F^2}{2}, \quad D_1 = 0, \quad A_3 = \frac{C_F^2}{2},\nonumber \\ A_2&= \frac{3}{4}C_F^2 + \frac{11}{24}C_F C_A -\frac{1}{6}C_F n_F T_R, \nonumber \\ A_1&= \bigg (\frac{1}{4}-\frac{\pi ^2}{12}\bigg ) C_F^2 + \bigg ( \frac{67}{72}-\frac{\pi ^2}{24} \bigg )C_F C_A -\frac{5}{18} C_F n_F T_R, \nonumber \\ B_4&= \frac{C_F^2}{8}, \quad B_3 = \frac{3}{8}C_F^2 + \frac{11}{96}C_F C_A -\frac{1}{24} C_F n_F T_R, \nonumber \\ B_2&=\! \bigg (\frac{13}{32}\!-\!\frac{\pi ^2}{48}\bigg ) C_F^2 \!+\! \bigg (\frac{83}{144}\!-\!\frac{\pi ^2}{96}\bigg )C_F C_A \!-\!\frac{7}{36} C_F n_F T_R, \nonumber \\ B_1&= \bigg (\frac{15}{64}-\frac{\pi ^2}{8} + \frac{2}{3}\zeta _3\bigg ) C_F^2 + \bigg (\frac{1357}{1728} \nonumber \\&\quad + \!\frac{11}{576}\pi ^2 \!-\!\frac{13}{16}\zeta _3\bigg )C_F C_A \!+\! \bigg (-\!\frac{101}{432} \!-\!\frac{\pi ^2}{144}\bigg )C_F n_F T_R. \end{aligned}$$

The coefficients $A_0$, $B_0$ and $D_0$ depend on the recombination scheme. In the *E*-scheme they read

52 $$\begin{aligned} D_0^E&= \left( \frac{\ln ^2(2)}{2}-\frac{\pi ^2}{6} \right) C_F^2, \nonumber \\ A_0^E&= -0.17976(1)~C_F^2 -2.20169(6)~C_F C_A \nonumber \\&\quad -0.12794(2)~C_F n_F T_R,\nonumber \\ B_0^E&= 4.514(1)~C_F^2 -0.2997(5)~C_F C_A\nonumber \\&\quad -0.2210(1)~C_F n_F T_R, \end{aligned}$$

and in the WTA scheme we find

53 $$\begin{aligned} D_0^{\textrm{WTA}}&= -\frac{\pi ^2}{12}C_F^2, \nonumber \\ A_0^{\textrm{WTA}}&= 2.01322(9)~C_F^2 -2.64831(2)~C_F C_A \nonumber \\&\quad -0.0766(1)~C_F n_F T_R, \nonumber \\ B_0^{\textrm{WTA}}&= 8.3346(8)~C_F^2 -1.7774(3)~C_F C_A \nonumber \\&\quad -0.0735(1)~C_F n_F T_R. \end{aligned}$$

The results in Eqs. (50)-(53) can be checked in several ways. We verified that the poles of the squared jet function reproduce those of the bare hard function for dijet production (i.e. the squared quark form factor [78]). In this case, the subtracted soft contribution is indeed finite as $\epsilon \rightarrow 0$ [69]. The coefficients of the logarithmic terms are in general observable dependent and cannot be compared directly with existing results. Their values, however, are directly relevant when the jet function is used to carry out NNLO computations with slicing schemes [69]. For completeness, we finally report also the corresponding coefficients for the observable-independent endpoint contribution, i.e., the contribution to the jet function from the first term in Eq. (42). They read

54 $$\begin{aligned} \begin{aligned} D_{2}^{\textrm{EP}}&=\frac{1}{2} C_F^2,\quad D_{1}^{\textrm{EP}}=0,\quad D_{0}^{\textrm{EP}}=-\frac{\pi ^2}{12}C_F^2,\\ A_{3}^{\textrm{EP}}&=\frac{1}{2}C_F^2,\quad A_{2}^{\textrm{EP}}=\frac{3}{4}C_F^2,\\ A_{1}^{\textrm{EP}}&=C_F^2\left( \frac{1}{4}-\frac{\pi ^2}{12}\right) ,\quad A_{0}^{\textrm{EP}}=-1.6343868(8)~C_F^2,\\ B_{4}^{\textrm{EP}}&=\frac{1}{8}C_F^2,\quad B_{3}^{\textrm{EP}}=\frac{3}{8}C_F^2,\\ B_{2}^{\textrm{EP}}&=C_F^2\left( \frac{23}{32}-\frac{5 \pi ^2}{48}\right) ,\quad B_{1}^{\textrm{EP}}=0.06315(3)~C_F^2\, ,\\ B_{0}^{\textrm{EP}}&= 0.4688(1)~C_F^2. \end{aligned} \end{aligned}$$

In Eqs. (52–54), alongside the numerical results, we quote the integration error. The observable-dependent subtracted contributions, which involve four-dimensional integrals, are computed with a dedicated Fortran code using Cuba [79], while the endpoint contribution coefficients are evaluated with Mathematica.

## Summary

The precise description of jet production processes requires observables capable of efficiently encoding the dynamics of the energy flow in hadronic final states. Transverse-momentum ($k_T$) like observables are shape variables that scale as the transverse momentum in the limit where the radiation becomes soft and collinear to the jet direction, thereby naturally capturing the leading singular behaviour of the multijet matrix elements.

In the region where additional radiation is inhibited, the multijet cross section is described in terms of hard, soft, beam, and jet functions. This organisation of the perturbative series remains useful even when a factorisation theorem for the observable under consideration is not available. Jet functions describe the collinear dynamics of partons inside a jet, and their perturbative evaluation for $k_T$-like variables is complicated by the necessity to account for the clustering history.

In this paper we have presented a semi-numerical approach for the computation of the NNLO quark jet function for $k_T$-like variables in $e^+e^-$ collisions. Our method can be applied to any observable in the class (1). The jet function is obtained by integrating collinear splitting kernels over the collinear phase space, and, at NNLO, it receives contributions from real and virtual terms. We have outlined the main steps of their NNLO computation in full generality. The calculation is affected by rapidity divergences, and we have regulated them by using a time-like auxiliary vector in the definition of collinear momentum fractions. We have presented explicit results for a variable that smoothly captures the $n+ 1$ to *n* jet transition in $e^+e^-$ collisions, both in the *E*-scheme and WTA scheme. The calculation can be easily adapted to other $k_T$-like variables. The results we have presented provide a building block for possible resummed computations for $k_T$ observables and are directly relevant for NNLO calculations of multijet cross sections at high-energy colliders when these observables are used as slicing variables [68]. First results in this direction will be presented elsewhere [69]. Our results may also be relevant in the matching of fixed-order computations to parton-shower simulations.

## Data Availability

This manuscript has no associated data. [Author’s comment: Data sharing not applicable to this article as no datasets were generated or analysed during the current study.]

## Distributional expansion for the observable-dependent contribution

For the observable-dependent (subtracted) part of the $C_F^2$ contribution to the jet function we need to expand the factor $1/(z_{1,N}\tilde{z}_{2,N})$ (see Eq. (40)) in the limit $k^2_{1,\perp },k^2_{23,\perp }\rightarrow 0$ at fixed values of $k^2_{1,\perp }/k^2_{23,\perp }$. In order to do so, we can use the distributional expansion

55 $$\begin{aligned}  &   \int _0^1 dz_1 \int _0^1 dz_2 \frac{z_1}{z_1^2+y^2 \lambda ^2}\frac{z_2}{z_2^2+ \frac{\lambda ^2}{(1-z_1)^2}}p(z_1,z_2)\nonumber \\  &   \quad =\int _0^1 dz_1 \int _0^1 dz_2 \frac{1}{z_1 z_2}\left( p(z_1,z_2)-p_s\left( \frac{z_1}{z_2}\right) \right. \nonumber \\  &   \qquad \left. -p_{s_1}(z_2)-p_{s_2}(z_1) +p_{s_1,s_2}+p_{s_2,s_1}\right) \nonumber \\  &   \qquad +\int _0^1 \frac{dz_1}{z_1}\log \left( \frac{1-z_1}{\lambda }\right) \left( p_{s_2}(z_1)-p_{s_2,s_1}\right) \nonumber \\  &   \qquad -\log (\lambda y)\int _0^1 \frac{dz_2}{z_2} \left( p_{s_1}(z_2)-p_{s_1,s_2}\right) \nonumber \\  &   \qquad +\int _0^1 \frac{dt}{t}\Biggl (\frac{y^2 \log \left( \frac{y}{t}\right) \left( p_s(t)-p_{s_1,s_2}\right) }{t^2-y^2}\nonumber \\  &   \qquad +\frac{\left( \log \left( \frac{1}{t}\right) -t^2 y^2 \log (y)\right) \left( p_s\left( \frac{1}{t}\right) -p_{s_2,s_1}\right) }{t^2 y^2-1}\nonumber \\  &   \qquad -\log (\lambda ) \left( p_s\left( \frac{1}{t}\right) +p_s(t)-p_{s_1,s_2}-p_{s_2,s_1}\right) \Biggr )\nonumber \\  &   \qquad +\frac{1}{4} \text {Li}_2\left( 1-\frac{1}{y^2}\right) \left( p_{s_1,s_2}-p_{s_2,s_1}\right) \nonumber \\  &   \qquad -\frac{1}{2} \log (\lambda y) \left( \log (y) \left( p_{s_2,s_1}-p_{s_1,s_2}\right) -\log (\lambda )\right. \nonumber \\  &   \qquad \left. \left( p_{s_1,s_2}+p_{s_2,s_1}\right) \right) \nonumber \\  &   \qquad -\frac{1}{6} \pi ^2 p_{s_2,s_1}+{\mathcal {O}}(\lambda ), \end{aligned}$$

where $p(z_1,z_2)$ is an integrable function with well-defined limits

56 $$\begin{aligned} \begin{aligned} p_{s_1}(z_2)&=\lim _{z_1\rightarrow 0}p(z_1,z_2)\quad \quad p_{s_2}(z_1)=\lim _{z_2\rightarrow 0}p(z_1,z_2)\\ p_{s}(t)&=\lim _{\lambda \rightarrow 0}p(\lambda t,\lambda )\\ p_{s_1,s_2}&=\lim _{z_2\rightarrow 0}\lim _{z_1\rightarrow 0}p(z_1,z_2)=\lim _{t\rightarrow 0}p_{s}(t)\\ p_{s_2,s_1}&=\lim _{z_1\rightarrow 0}\lim _{z_2\rightarrow 0}p(z_1,z_2)=\lim _{t\rightarrow 0}p_{s}\left( \frac{1}{t}\right) . \end{aligned} \end{aligned}$$

## Footnotes

1 A similar (though not exactly equivalent) regularisation procedure was used in Ref. [64], whereas Ref. [60] uses a *space-like* auxiliary vector *N*.

2 This assumption is not essential for the construction of the method but leads to some useful simplifications.

3 We note that the one-loop splitting kernel ${{\hat{P}}}^{(1)}_{gq}(z)$ in Eq. (19) behaves as $z^{-1-\epsilon }$ in the $z\rightarrow 0$ limit but the $z^{-\epsilon }$ factor is compensated by a $z^{\epsilon }$ that originates from the $s_{12}^{-\epsilon }$ prefactor in Eq. (6).

4 The $z_{N}$-regulated NLO jet function for the case of the standard distance used in the $k_T$ algorithm can be found in Ref. [53].

## Code Availability Statement

This manuscript has no associated code/software. [Author’s comment: Code/Software sharing not applicable to this article as no code/software was generated or analysed during the current study.]
